# Insulin receptor substrate 2 (IRS2) deficiency delays liver fibrosis associated with cholestatic injury

**DOI:** 10.1242/dmm.038810

**Published:** 2019-07-16

**Authors:** Andrea Villar-Lorenzo, Patricia Rada, Esther Rey, Patricia Marañón, Ana I. Arroba, Beatriz Santamaría, Jorge Sáiz, Francisco J. Rupérez, Coral Barbas, Carmelo García-Monzón, Ángela M. Valverde, Águeda González-Rodríguez

**Affiliations:** 1 Instituto de Investigaciones Biomédicas Alberto Sols (CSIC-UAM), 28029 Madrid, Spain; 2 Centro de Investigación Biomédica en Red de Diabetes y Enfermedades Metabólicas Asociadas (CIBERDEM), Instituto de Salud Carlos IIII, 28029 Madrid, Spain; 3 Unidad de Investigación Hepática, Hospital Universitario Santa Cristina, Instituto de Investigación Sanitaria del Hospital Universitario de La Princesa, 28009 Madrid, Spain; 4 Centre for Metabolomics and Bioanalysis (CEMBIO), Faculty of Pharmacy, Universidad San Pablo CEU, Campus Monteprincipe, Boadilla del Monte, 28668, Madrid, Spain; 5 Centro de Investigación Biomédica en Red de Enfermedades Hepáticas y Digestivas (CIBEREHD), Instituto de Salud Carlos IIII, 28029 Madrid, Spain

**Keywords:** IRS2, Fibrosis, Bile acids, Cholestatic injury, Hepatic stellate cells, IGF1

## Abstract

Insulin receptor substrate 2 (IRS2) is a key downstream mediator of insulin and insulin-like growth factor 1 (IGF1) signalling pathways and plays a major role in liver metabolism. The aim of this study was to investigate whether IRS2 had an impact on the hepatic fibrotic process associated with cholestatic injury. Bile duct ligation (BDL) was performed in wild-type (WT) and *Irs2*-deficient (IRS2KO) female mice. Histological and biochemical analyses, together with fibrogenic and inflammatory responses were evaluated in livers from mice at 3, 7 and 28 days following BDL. We also explored whether activation of human hepatic stellate cells (HSCs) induced by IGF1 was modulated by IRS2. IRS2KO mice displayed reduced disruption of liver histology, such hepatocyte damage and excess deposition of extracellular matrix components, compared with WT mice at 3 and 7 days post-BDL. However, no histological differences between genotypes were found at 28 days post-BDL. The less pro-inflammatory profile of bile acids accumulated in the gallbladder of IRS2KO mice after BDL corresponded with the reduced expression of pro-inflammatory markers in these mice. Stable silencing of *IRS2* or inhibition of ERK1/2 reduced the activation of human LX2 cells and also reduced induction of MMP9 upon IGF1 stimulation. Furthermore, hepatic MMP9 expression was strongly induced after BDL in WT mice, but only a slight increase was found in mice lacking IRS2. Our results have unravelled the signalling pathway mediated by IGF1R–IRS2–ERK1/2–MMP9 as a key axis in regulating HSC activation, which might be therapeutically relevant for targeting liver fibrosis.

## Abstract (precis)

**Summary:** IRS2 is a key mediator of IGF1R signalling in hepatic stellate cell activation in cholestatic liver injury.

## INTRODUCTION

In chronic cholestatic diseases such as primary biliary cholangitis and primary sclerosing cholangitis, the accumulation of bile acids in the hepatobiliary system induces toxic effects in liver cells leading to hepatic damage and inﬂammation with progression to ﬁbrosis, cirrhosis and eventually liver failure (Fickert and Wagner, 2017). In the early stages of the cholestasis process, hydrophobic bile acids induce hepatocyte cell injury and are responsible for the inflammatory and fibrogenic responses of non-parenchymal liver cells (NPCs) (Czaja, 2014; Lee and Friedman, 2011). This hepatocyte damage increases the expression of growth factors, cytokines and chemokines, which boost the inflammatory response and induce fibrogenesis by activating hepatic stellate cells (HSCs) (Tsuchida and Friedman, 2017). Activated HSCs are the primary source of the extracellular matrix (ECM) components that accumulate in the liver during fibrosis as a result of upregulation of proteins including α-smooth muscle actin (αSMA or ACTA2) and interstitial collagens such as collagen type 1 (COL1A1). Moreover, HSCs contribute to the inflammatory process associated with fibrosis by enhancing the secretion of pro-inflammatory cytokines from resident immune cells of the liver (Koyama and Brenner, 2017; Tsuchida and Friedman, 2017). Apart from ECM synthesis and deposition, matrix degradation also participates in the pathological process of liver fibrosis. In this context, the matrix metalloproteinases (MMPs) play a key role in the maintenance of the balance between the synthesis and degradation of collagen fibres (Duarte et al., 2015; Lichtinghagen et al., 1995). After an acute liver injury, changes that occur in the ECM are transient and the hepatic architecture is completely restored. However, in chronic liver damage, the unresolved immune response persists and ECM proteins accumulate in the hepatic tissue as they cannot be degraded by MMPs. Because of this, the hepatic parenchyma is progressively replaced by fibrotic scar tissue (Hernandez-Gea and Friedman, 2011; Pinzani and Luong, 2018). In addition to transforming growth factor β (TGFβ), different cytokines and growth factors contribute to HSC activation. An early study by Svegliati-Baroni and colleagues (Svegliati-Baroni et al., 1999) provided strong evidence for the role of the insulin-like growth factor 1 (IGF1)/IGF1 receptor (IGF1R) system in promoting HSC mitogenesis and COL1A1 synthesis.

Insulin receptor substrate 2 (IRS2) is one of the key mediators of insulin and IGF1 signalling downstream of their activated receptors, and it regulates a variety of processes, including metabolism, cellular growth, development and survival (White, 2014). *Irs2*-deficient (IRS2KO) male mice display basal hyperglycaemia, systemic insulin resistance, glucose intolerance, pancreatic β cell dysfunction due to impaired IGF1R-mediated mitogenic signalling, and eventually, type 2 diabetes, with most mice dying at ∼16 weeks of age because of diabetic complications (Kubota et al., 2000; Withers et al., 1998). In the liver, IRS2 deficiency impairs the insulin response in the activation of phosphatidylinositol 3-kinase (PI3K)/Akt pathway and in the inhibition of gluconeogenic gene expression (González-Rodriguez et al., 2010, 2015; Kubota et al., 2000; Withers et al., 1998). In contrast, IRS2KO females did not develop hyperglycaemia nor did they show alterations in insulin signalling or hepatic gluconeogenesis (Burks et al., 2000), indicating a gender-dependent diabetic phenotype in this mouse model.

Although multiple studies have defined IRS2 as a critical node of hepatic insulin signalling (White, 2014), its role in other physiological processes in the liver that are not related to insulin action has been poorly studied. The lack of IRS2 in neonatal hepatocytes accelerates cell death upon serum withdrawal (Valverde et al., 2004), suggesting that IRS2 participates in the maintenance of the balance between cell death and survival. Taking this previous research into account, the aim of the present study was to investigate whether IRS2 exerts any effect on the fibrotic process in livers with cholestatic injury. To achieve this goal, bile duct ligation (BDL) was conducted in wild-type (WT) and IRS2KO female mice in order to avoid the effects mediated by the profound metabolic alterations of the male mice. We explored the inflammatory and fibrogenic responses triggered after BDL, as well as whether or not IGF1-mediated activation of LX2 cells, a human HSC line, is modulated by IRS2.

## RESULTS

### Evolution of cholestatic liver injury in wild-type and *Irs2*-deficient mice

**Fig. 1.**
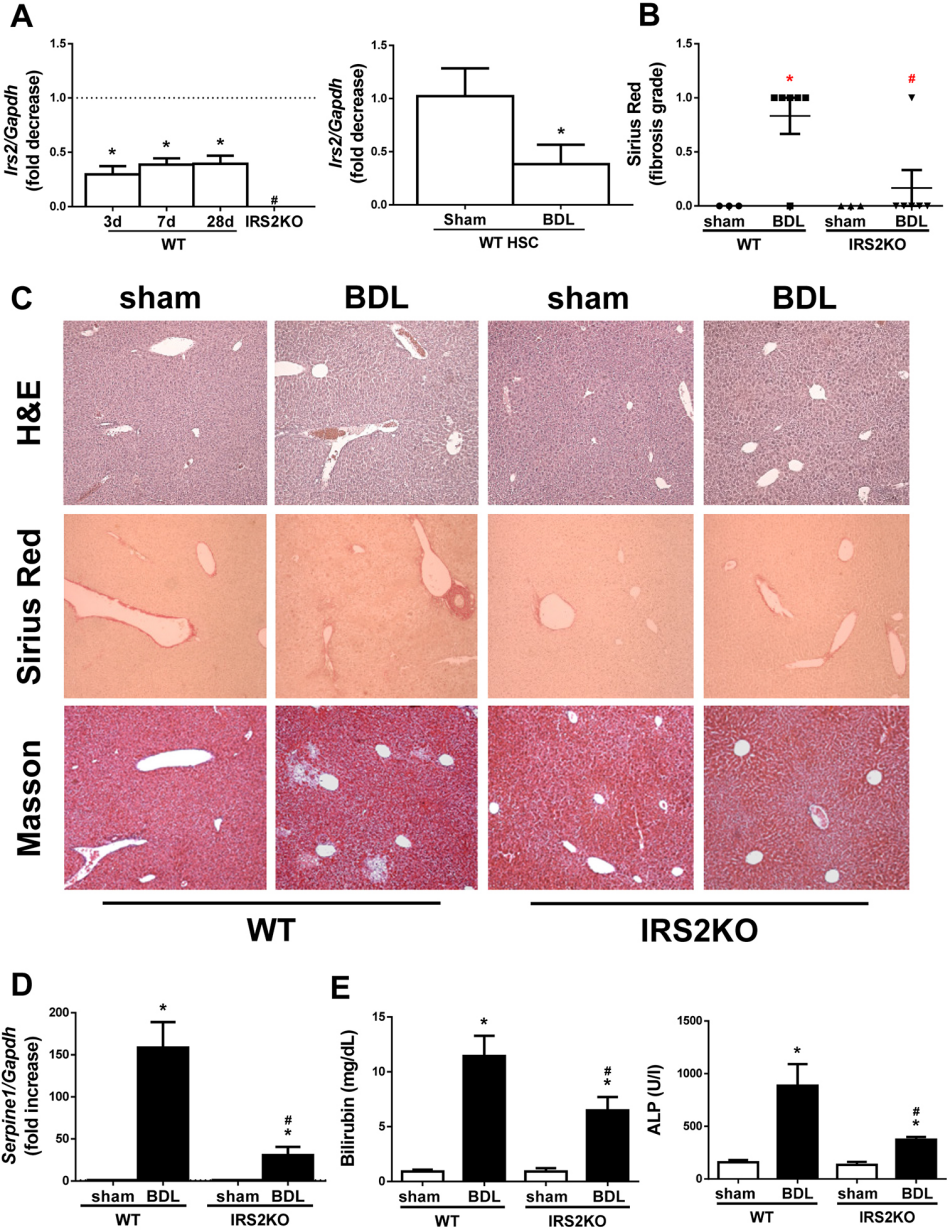
***Irs2* expression** **after cholestatic damage and analysis of histological alterations and liver biliary damage markers in wild-type and *Irs2*-deficient mice at 3 days post-bile duct ligation (BDL).** (A) *Irs2* mRNA levels determined by RT-qPCR. (B) Sirius Red fibrosis score. (C) Representative images (20×) of liver sections after haematoxylin and eosin (H&E), Sirius Red and Masson's trichrome staining. (D) *Serpine1* mRNA levels determined by RT-qPCR. (E) Serum bilirubin and alkaline phosphatase (ALP) levels. Wild-type (WT) and *Irs2*-deficient (IRS2KO) female mice were submitted to BDL or sham operated and analyzed at the indicated times (3 days after BDL in B–E). Statistical analysis was carried out by one-way ANOVA with Bonferroni post-test: *P<0.05, BDL versus sham; ^#^*P*<0.05, IRS2KO versus WT (*n*=8 animals per condition).

In order to investigate the impact of *Irs2* knockdown in liver fibrosis, cholestatic liver injury was induced by BDL in WT and IRS2KO female mice. Figure S1 shows no differences in food intake between the two genotypes. Interestingly, in WT mice, *Irs2* gene expression decreased after cholestatic injury in both livers and isolated HSCs (Fig. 1A). Accordingly, *IRS2* expression also decreased in activated human LX2 HSCs (Fig. S2). Upon histological examination at 3 days after BDL, there was evidence of ECM deposition around the reactive bile ducts in livers from WT mice, as demonstrated by both Masson's trichrome and Sirius Red staining (Fig. 1B,C). Moreover, mRNA expression of *Serpine1*, encoding the plasminogen activator inhibitor type I (PAI1), a well-characterized fibrotic marker, was also elevated after BDL in livers from WT mice (Fig. 1D). As expected, serum levels of cholestatic liver damage markers such as bilirubin and alkaline phosphatase (ALP) were significantly higher than the corresponding values of control (sham operated) mice (Fig. 1E). Interestingly, *Irs2*-deficient females exhibited less BDL-induced liver damage as reflected by almost normal liver histology and reduced elevation of hepatic *Serpine1* mRNA, as well as lower serum bilirubin and ALP levels when compared with the WT response.

**Fig. 2.**
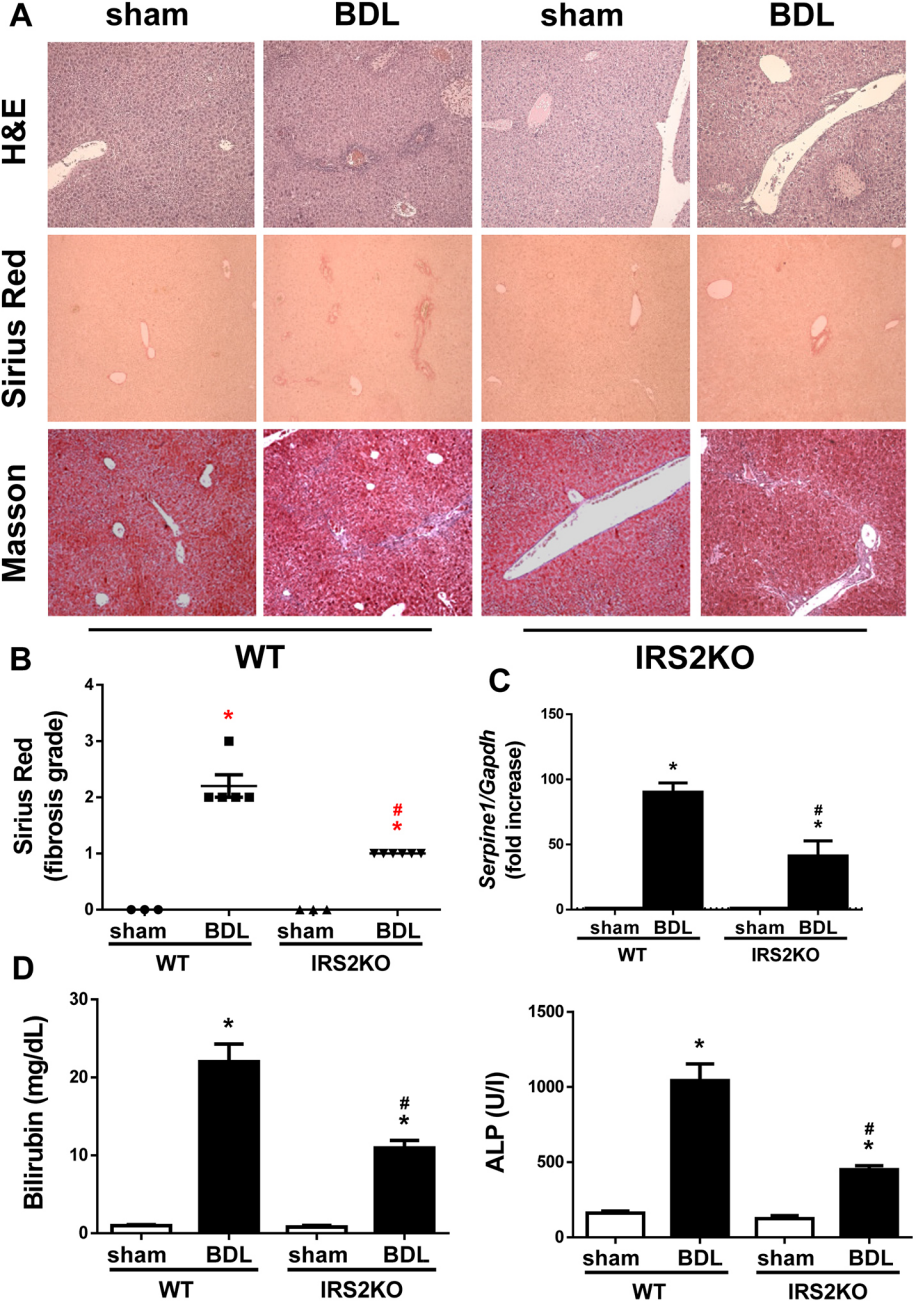
**Histological alterations and liver biliary damage in wild-type and *Irs2*-deficient mice at 7 days post-BDL.** (A) Representative images (20×) of H&E, Sirius Red and Masson's trichrome staining of liver sections. (B) Sirius Red fibrosis score. (C) *Serpine1* mRNA levels determined by RT-qPCR. (D) Serum bilirubin and alkaline phosphatase (ALP) levels. Wild-type (WT) and *Irs2*-deficient (IRS2KO) female mice were submitted to BDL or sham operated and analyzed 7 days after surgery. Statistical analysis was carried out with one-way ANOVA and Bonferroni post-test: *P<0.05, BDL versus sham; ^#^*P*<0.05, IRS2KO versus WT (*n*=6–8 animals per condition).

**Fig. 3.**
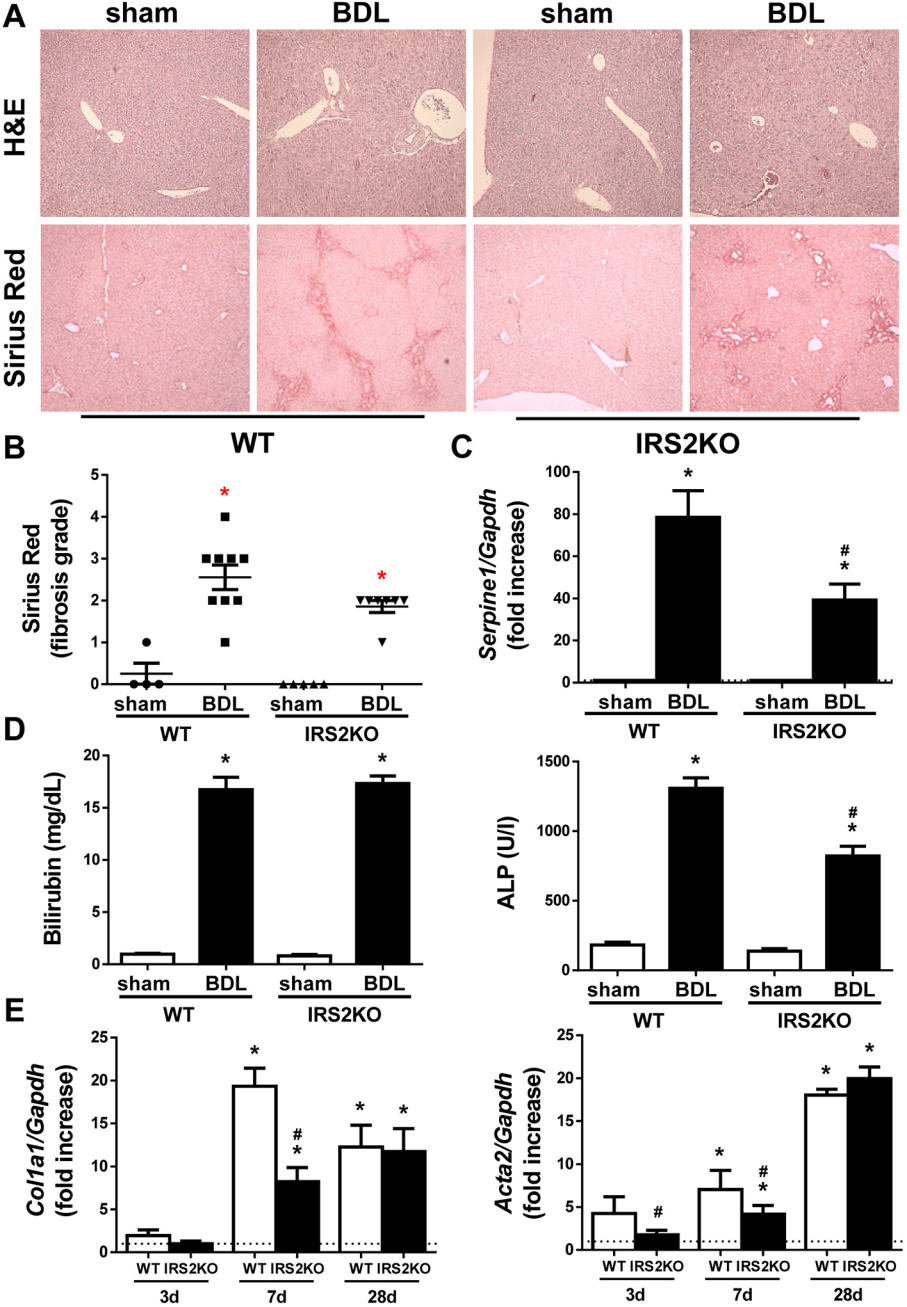
**Profibrogenic markers in livers from wild-type and *Irs2*-deficient mice** **after BDL.** (A) Representative images (20×) of liver sections after H&E and Sirius Red staining. (B) Sirius Red fibrosis score. (C) *Serpine1* mRNA levels determined by RT-qPCR. (D) Serum bilirubin and alkaline phosphatase (ALP) levels. (E) *Col1a1* and *Acta2* mRNA levels determined by RT-qPCR. Wild-type (WT) and *Irs2*-deficient (IRS2KO) female mice were submitted to BDL or sham operated and analyzed at the indicated time periods (28 days after BDL in A–D). Statistical significance was assessed by one-way ANOVA with Bonferroni post-test: *P<0.05, BDL versus sham; ^#^*P*<0.05, IRS2KO versus WT (*n*=6–8 animals per condition).

A similar analysis performed in liver samples collected 7 days following BDL revealed that hepatic fibrogenesis continued its expansion in WT mice, a process monitored by the visualization of bile infarcts in the hepatic tissue in addition to increased collagen deposition (Fig. 2A,B), *Serpine1* mRNA expression (Fig. 2C) and serum bilirubin and ALP (Fig. 2D). Notably, the progression of fibrotic damage was significantly attenuated in livers from mice lacking IRS2, as ECM deposition around the reactive bile ducts (observed in WT mice 3 days post-BDL) became evident after 7 days. In addition, at this time, the induction of cholestatic damage and fibrogenic markers was lower than it was in their WT counterparts. Surprisingly, the differences in the time frame of the fibrotic process observed between the two genotypes of mice in liver histology and serum bilirubin were not so evident at 28 days post-BDL (Fig. 3A,B,D and Fig. S3), time at which fibrosis process was established in both genotypes, although serum ALP and hepatic *Serpine1* content was less elevated in IRS2KO mice compared with the WT mice (Fig. 3C,D).

At the molecular level, hepatic mRNA expression *Col1a1* and *Acta2* – two key drivers of the fibrogenic process – was increased in a time-dependent manner after BDL in WT animals (Fig. 3E); meanwhile, the lack of IRS2 delayed these elevations at early time periods post-BDL (3 and 7 days).

### Differences in the bile acid content in the gallbladder of wild-type and *Irs2*-deficient mice after BDL

**Fig. 4.**
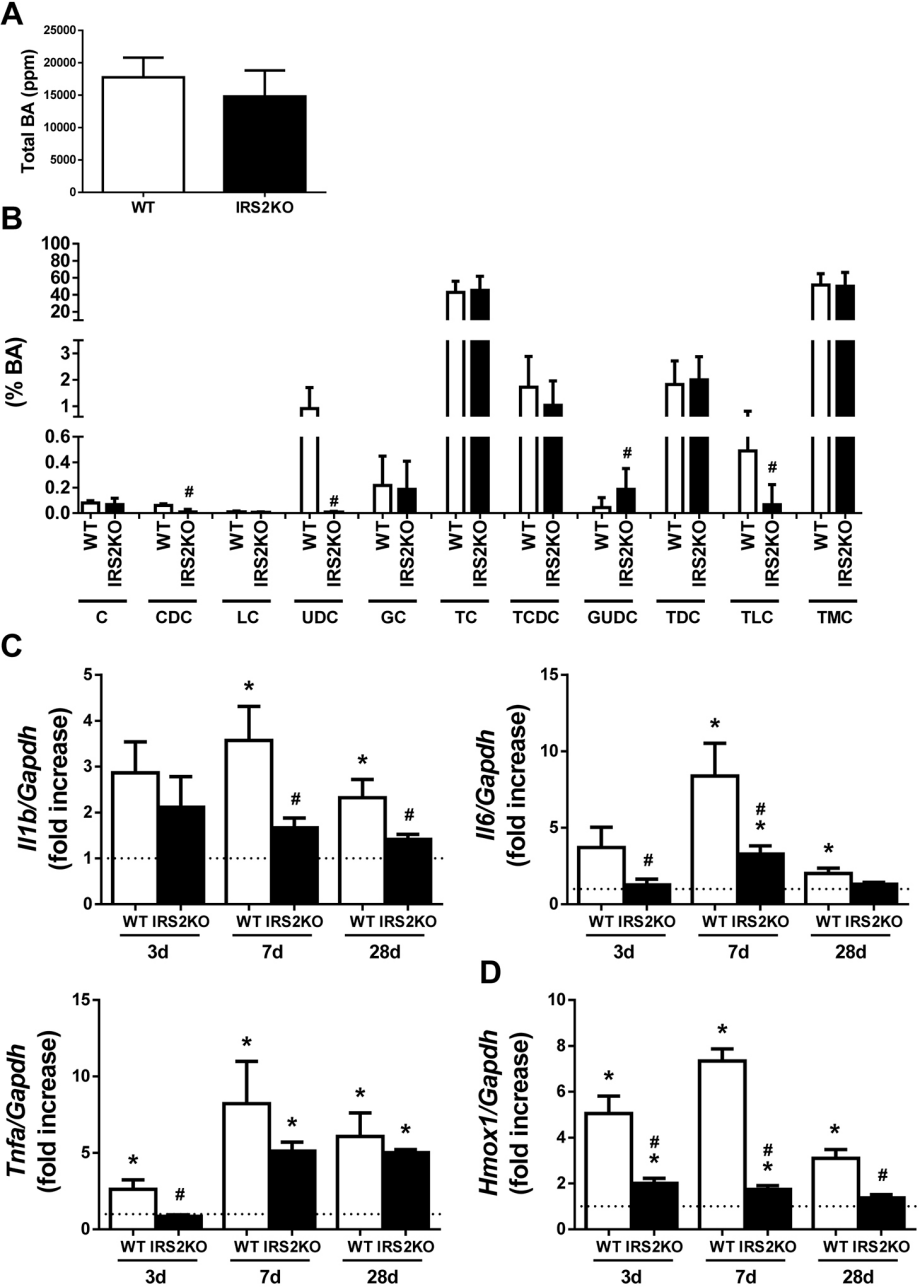
**Proinflammatory markers in livers from wild-type and *Irs2*-deficient mice** **after BDL.** (A) Total bile acid (BA) content accumulated in the gallbladder (ppm). (B) Composition of bile liquid accumulated in the gallbladder (%). C, cholic acid; CDC, chenodeoxycholic acid; LC, lithocholic acid; UDC, ursodeoxycholic acid; GC, glycocholic acid; TC, taurocholic acid; TCDC, taurocholicdeoxycholic acid; GUDC, glycoursodeoxycholic acid; TDC, taurodeoxycholic acid; TLC, taurolithocholic acid; TMC, tauroalphamuricholic. *Il1b*, *Il6* and *Tnfa* (C), and *Hmox1* (D) mRNA levels determined by RT-qPCR. Wild-type (WT) and *Irs2*-deficient (IRS2KO) female mice were submitted to BDL or sham operated and analyzed at the indicated time-periods (28 days after BDL in A and B). Statistical significance was assessed using unpaired two-tailed Student's *t*-test (A,B) or by one-way ANOVA with Bonferroni post-test (C,D): *P<0.05, BDL versus sham; ^#^*P*<0.05, IRS2KO versus WT (*n*=6–8 animals per condition).

Next, we investigated if the delay in the progression of the fibrotic process induced by biliary damage in IRS2KO mice could be due to a different profile of bile acids accumulated in the gallbladder after BDL. The analysis by mass spectrometry did not reveal differences between genotypes in the total amount of bile acids accumulated in the gallbladder at 28 days post-BDL (Fig. 4A). However, a lower percentage of three specific bile acids – chenodeoxycholic acid (CDC), ursodeoxycholic acid (UDC) and taurolitocholic acid (TLC) – was detected in BDL-operated IRS2KO mice compared with BDL-operated WT mice. On the other hand, the lack of IRS2 increased the percentage of glucoursodeoxycholic acid (GUDC), which is derived from UDC by conjugation with glycine (Fig. 4B).

### IRS2 deficiency delays the inflammatory process after BDL

In addition to liver damage caused by ECM deposition, cholestatic injury is characterized by focal inflammation and subsequent oxidative stress. Hepatic mRNA levels of pro-inflammatory markers such as *Il1b*, *Il6* and *Tnfa* were measured and IRS2 deficiency was found to decrease the induction of *IL1b* mRNA and retard the elevation of *Il6* and *Tnfa* mRNAs after BDL (Fig. 4C). Likewise, the expression of heme oxygenase 1 (*Hmox1*), one of the main target genes of the Nrf2-mediated antioxidant system, was induced in the livers of WT mice after BDL, an effect that was absent in IRS2KO mice (Fig. 4D).

Altogether, results in the BDL model suggest that IRS2 deficiency retards the progression of cholestasis-induced liver fibrosis, an effect that is likely due to a slow-down of HSC activation.

### IGF1 induces activation of LX2 cells

**Fig. 5.**
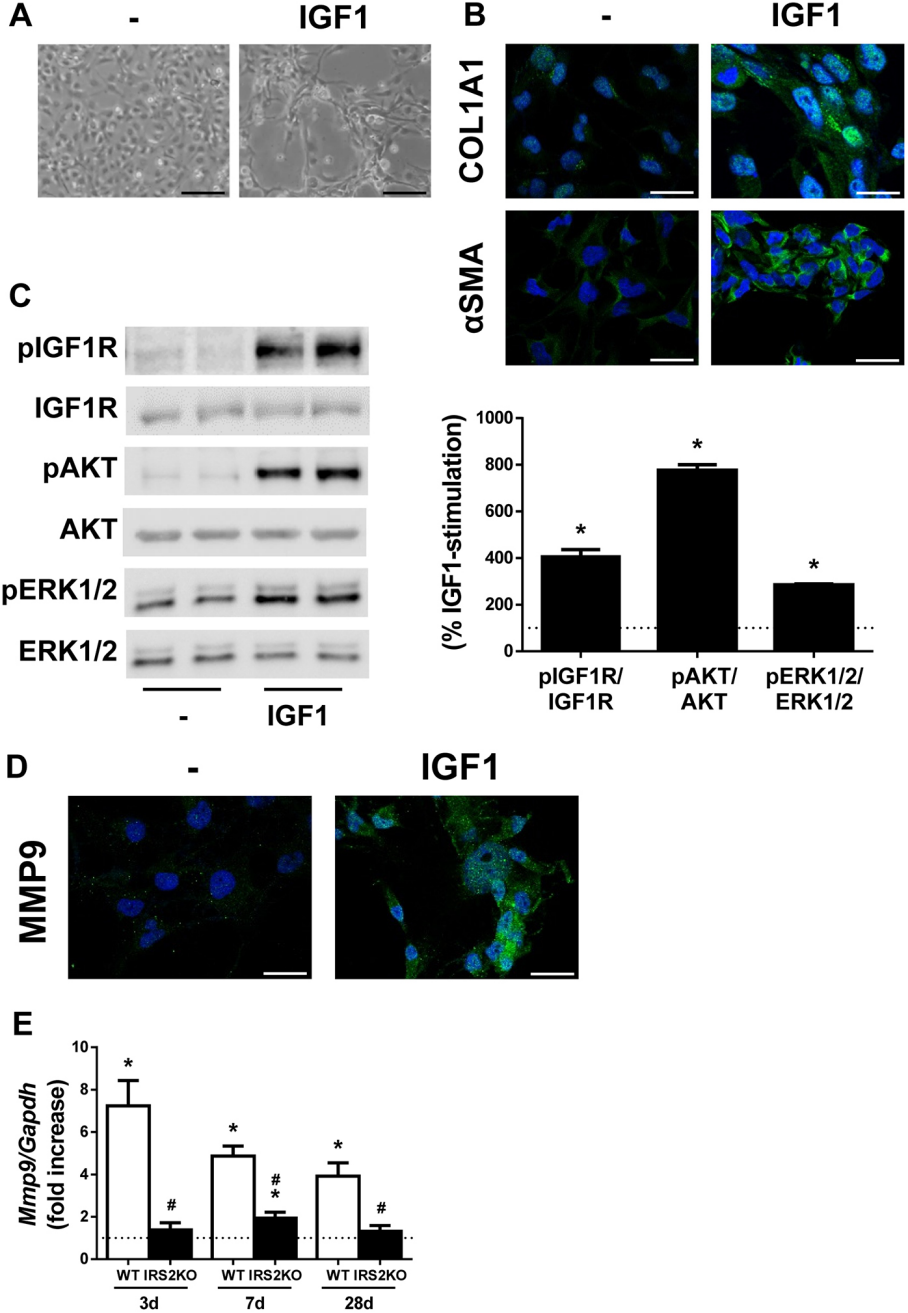
**IGF1 induces the activation of LX2 cells.** LX2 cells were treated with 10 nM IGF1 for 24 h (A,B,D) or 15 min (C). (A) Representative phase-contrast images of LX2 cells. (B) Representative images of LX2 cells after immunofluorescence staining for COL1A1 and αSMA. Nuclei are stained blue with DAPI. (C) Representative western blots with the indicated antibodies (left) and their quantification (right). Statistical significance was assessed by unpaired two-tailed Student's *t*-test: *P<0.05, IGF1 versus untreated cells (*n*=5 independent experiments performed in duplicate). (D) Representative images of LX2 cells after immunofluorescence staining for MMP9. Nuclei are stained blue with DAPI. (E) *Mmp9* mRNA levels determined by RT-qPCR. Wild-type (WT) and *Irs2*-deficient (IRS2KO) female mice submitted to BDL or sham operated and analyzed at the indicated time-periods. Statistical significance was assessed by one-way ANOVA and Bonferroni post-test: *P<0.05, BDL versus sham; ^#^*P*<0.05, IRS2KO versus WT (*n*=6–8 animals per condition). Scale bars: 100 μm (A) and 25 μm (B,D).

It has been reported that IGF1, like TGFβ, can induce HSC activation and proliferation (Svegliati-Baroni et al., 1999). As IRS2 is a key molecule in IGF1R-mediated signalling, we explored its potential role in mediating the activation of HSCs by IGF1. Firstly, we assessed the activation of LX2 cells by IGF1. The phase-contrast microscopy images revealed that LX2 cells treated with 10 nM IGF1 for 24 h showed morphological features of activated HSCs evolving into myofibroblast-like cells compared with the untreated cells (Fig. 5A). Also, increased expression of the HSC activation markers αSMA and COL1A1 in IGF1-stimulated LX2 cells was detected by immunofluorescence (COL1A1, 4.76±1.04-fold, *P*<0.05; αSMA, 4.90±0.38-fold, *P*<0.05) (Fig. 5B). Regarding the IGF1 intracellular signalling cascade, tyrosine phosphorylation of both IGF1R (Fig. 5C) and IRS1 (Fig. S4) was increased in IGF1-stimulated LX2 cells. Moreover, AKT and ERK1/2, two major IGF1R downstream targets, were also phosphorylated in IGF1-treated LX2 cells (Fig. 5C). Expression of MMP9 was also analyzed by immunofluorescence since it is an extracellular MMP overexpressed after activation of HSCs and whose expression is regulated by ERK1/2 (Loesch et al., 2010). As shown in Fig. 5D, MMP9 content was significantly higher in LX2 cells treated with IGF1 compared with the untreated cells (MMP9, 5.18±0.57-fold, *P*<0.05). Analysis of levels of mRNA encoding inflammatory mediators revealed that IGF1-induced activation of LX2 cells did not correlate with a pro-inflammatory effect in HSCs (Fig. S5).

### IRS2 deficiency reduces hepatic MMP9 expression induced by cholestatic damage

We analyzed the hepatic mRNA expression of MMP9 in WT and IRS2KO mice after BDL. As shown in Fig. 5E, it was significantly increased in WT mice 3 days post-BDL and reduced gradually over the following time periods analyzed, whereas *Mmp9* mRNA increased only slightly in IRS2KO mice at 7 days post-BDL. Interestingly, hepatic *Igf1* mRNA expression decreased after cholestatic damage in both WT and IRS2KO female mice (Fig. S6).

### IRS2 mediates the IGF1 signalling cascade that leads to activation of LX2 cells

**Fig. 6.**
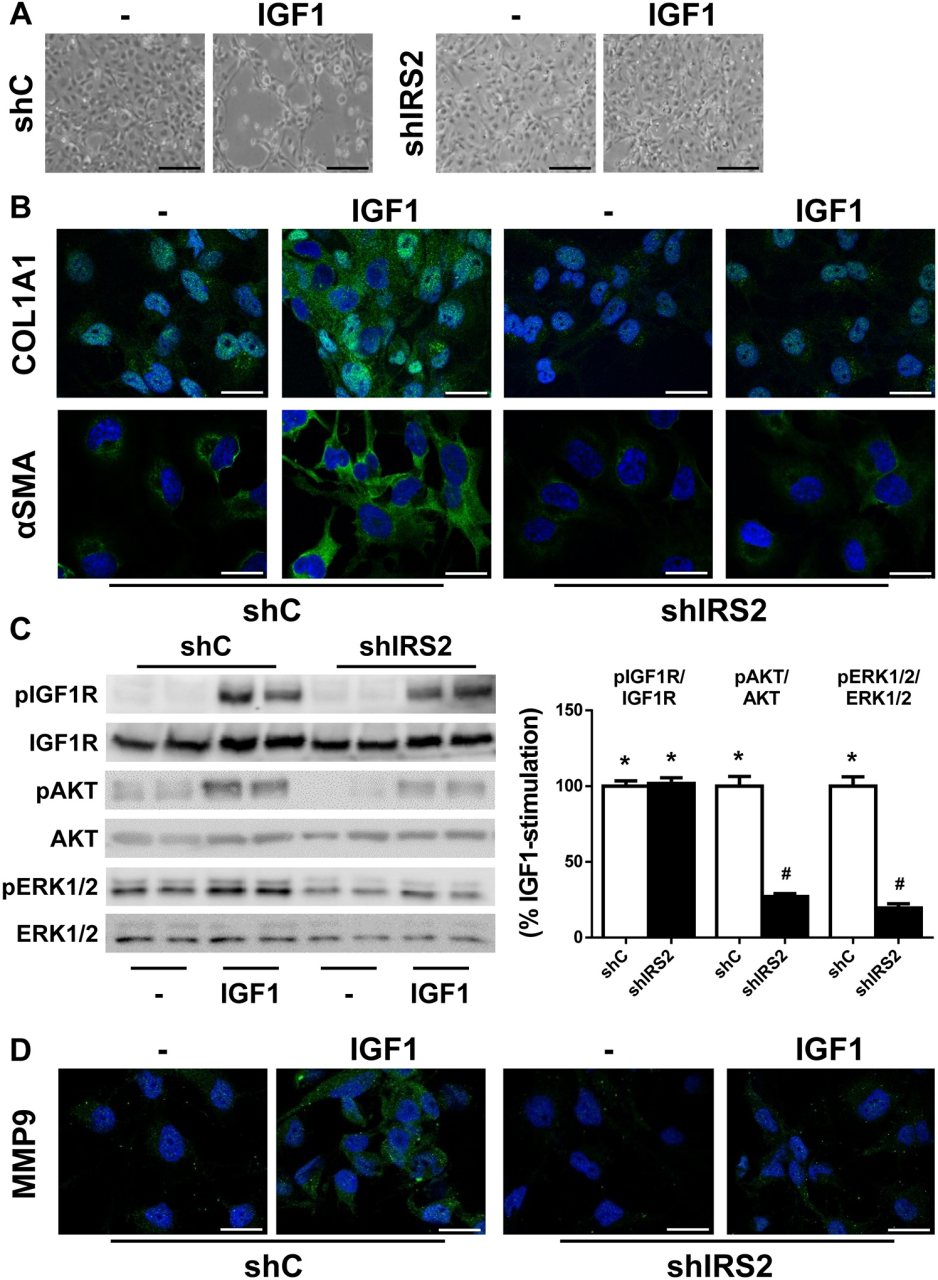
**Silencing of *IRS2*** **abolishes the activation of LX2 cells induced by IGF1.** LX2 cells infected with scrambled (shC) or IRS2 shRNA (shIRS2) lentiviral particles were treated with 10 nM IGF1 for 24 h (A,B,D) or 15 min (C). (A) Representative phase-contrast images of LX2 cells. (B) Representative images of LX2 cells after immunofluorescence staining for COL1A1 and αSMA. Nuclei are stained blue with DAPI. (C) Representative western blots with the indicated antibodies (left) and their quantification (right). (D) Representative images of LX2 cells after immunofluorescence staining for MMP9. Nuclei are stained blue with DAPI. Statistical significance was assessed by one-way ANOVA and Bonferroni post-test: *P<0.05, IGF1 versus untreated cells; ^#^*P*<0.05, shIRS2KO versus shC (*n*=4 independent experiments performed in duplicate). Scale bars: 100 μm (A) and 25 μm (B,D).

Next, we infected LX2 cells with scrambled (sh-control, shC) or IRS2 shRNA (shIRS2) lentiviral particles and, as Fig. S7 shows, a 75% reduction of *IRS2* mRNA levels was achieved. Stable silencing of *IRS2* significantly reduced IGF1-mediated activation of LX2 cells, as these cells preserved almost the same quiescent phenotype as the untreated cells and showed reduced expression of αSMA and COL1A1 (COL1A1: shC, 4.75±0.44-fold; shIRS2, 1.48±0.24-fold, *P*<0.05; αSMA: shC, 2.11±0.38-fold; shIRS2, 0.97±0.11-fold, *P*<0.05) (Fig. 6A,B). Moreover, IRS2 knockdown impaired the phosphorylation of AKT and ERK1/2, as well as the induction of MMP9 upon IGF1 stimulation (MMP9: shC, 7.23±1.93-fold; shIRS2, 1.63±0.16-fold, *P*<0.05) (Fig. 6C,D).

**Fig. 7.**
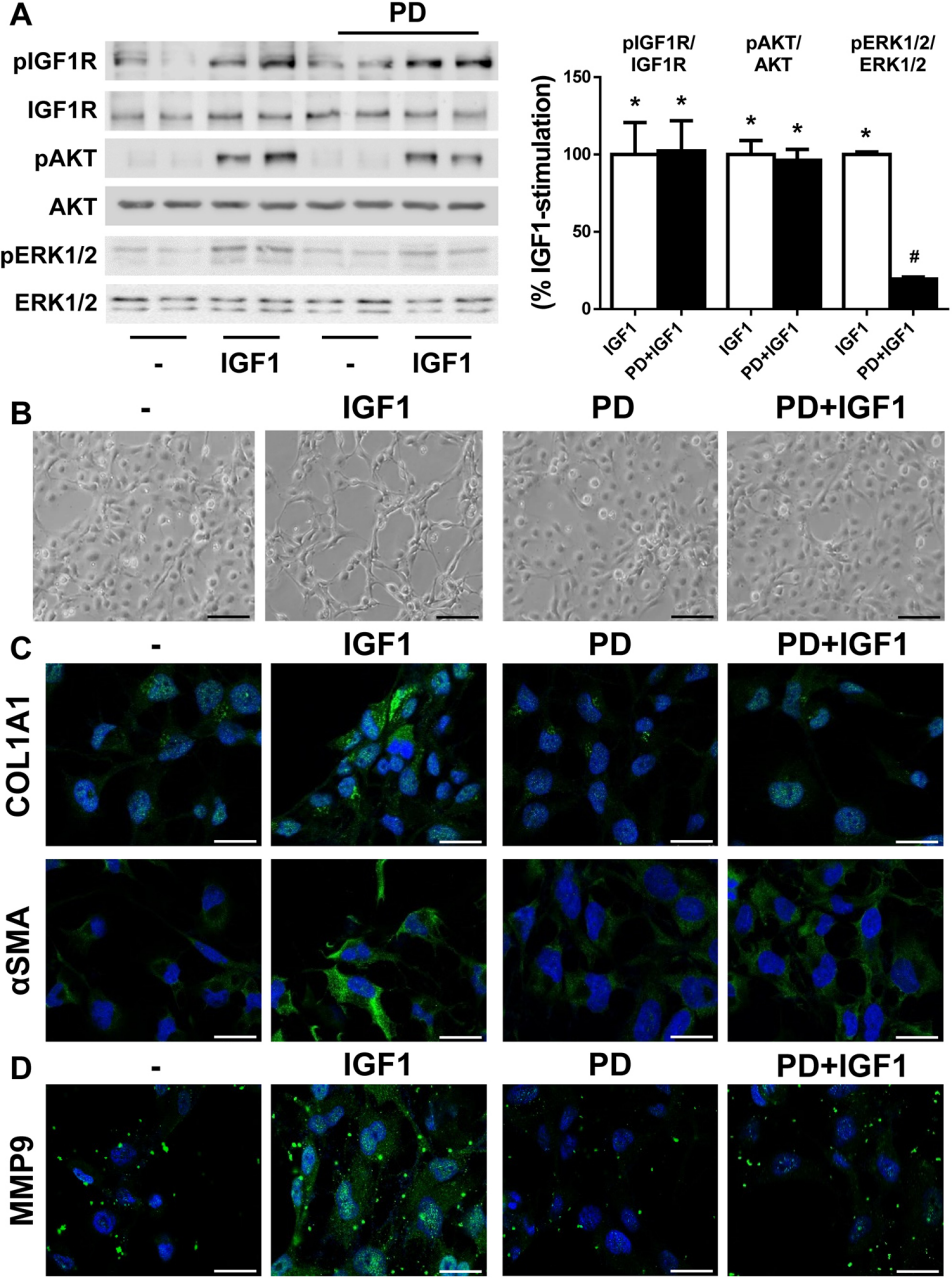
**ERK1/2 inhibition blocks the activation of LX2 cells induced by IGF1.** LX2 cells were pretreated with 20 µM PD98059 (PD) for 2 h prior to 10 nM IGF1 stimulation for 15 min (A) or 24 h (B–D). (A) Representative western blot with the indicated antibodies (left) with quantification (right). (B) Representative phase-contrast images of LX2 cells. (C,D) Representative images of LX2 cells after immunofluorescence staining for COL1A1 and αSMA (C), and MMP9 (D). Nuclei are stained blue with DAPI. Statistical significance was assessed using one-way ANOVA with Bonferroni post-test: *P<0.05, IGF1 versus untreated cells; ^#^*P*<0.05, PD-treated versus untreated cells (*n*=4 independent experiments performed in duplicate). Scale bars: 100 μm (B) and 25 μm (C,D).

To delineate the role of the IGF1R–ERK1/2–MMP9 axis in HSC activation, we treated LX2 cells with IGF1 together with PD98059, a well-known inhibitor of mitogen-activated protein kinase kinase (MEK). As expected, PD98059 inhibited ERK1/2 phosphorylation induced by IGF1 without altering IGF1R and AKT phosphorylation (Fig. 7A). Interestingly, pre-treatment with PD98059 impaired the activation of LX2 cells initiated by IGF1, which was visualized by the absence of a fibroblast-like phenotype under these conditions (Fig. 7B). Moreover, analysis of fibrogenic proteins by immunofluorescence staining showed reduced content of both αSMA and COL1A1 in IGF1-stimulated LX2 cells pretreated with PD98059 (COL1A1: no PD, 7.52±1.54-fold; PD, 1.70±0.19-fold, *P*<0.05; αSMA: no PD, 1.83±0.22 fold; PD, 0.86±0.04-fold, *P*<0.05) (Fig. 7C). Under these experimental conditions, we also checked MMP9 expression and, as shown in Fig. 7D, its induction in response to IGF1 decreased upon PD98059 pre-treatment (MMP9: no PD, 5.29±0.54-fold; PD, 1.52±0.09 fold, *P*<0.05). The inhibitor alone did not modify the phenotype or the expression of HSC activation markers of LX2 cells. Notably, inhibition of AKT with LY294002 did not change the morphological features of activated HSCs induced by IGF1 (Fig. S8).

## DISCUSSION

Liver fibrosis occurs as a healing process in response to liver damage underlying chronic liver diseases of diverse etiopathology, such as excessive alcohol consumption, hepatitis virus infection, steatosis, insulin resistance, as well as cholestatic injury. During injury to the hepatobiliary system, the accumulation and altered composition of bile exerts a toxic effect in the tissue. The fibrotic process begins with an early inflammatory response in order to counteract and resolve the underlying damage. However, when this primary response is not able to resolve the liver damage, the resulting chronic inflammatory environment triggers a fibrogenic process that involves alterations in the composition and distribution of the hepatic ECM components, in particular those caused by activation of the HSCs (Czaja, 2014; Higashi et al., 2017).

IRS2 coordinates insulin and IGF1 signalling cascades in different tissues, regulating a variety of processes, including metabolism, cellular growth, development and survival (White, 2014). In the present study, we analyzed for the first time the impact of IRS2 deficiency in the fibrotic process associated with cholestatic damage in the liver. As a preclinical model of cholestatic disease, we used BDL surgery in WT and *Irs2*-deficient female mice because, as indicated above, they develop only a moderate prediabetic metabolic phenotype compared with the severe diabetic phenotype of the male mice (Burks et al., 2000; Withers et al., 1998).

The results obtained in this study revealed a delay in the development and further progression of the fibrotic process associated with the biliary damage in the livers of IRS2KO mice after the BDL when compared with their corresponding WT counterparts. Histological analysis performed at 3 and 7 days post-BDL showed that the absence of IRS2 reduced the degree of hepatic fibrosis. Furthermore, at these same time periods, serum markers of liver and biliary damage, such as bilirubin and ALP, in conjunction with the hepatic expression of the fibrotic marker PAI1, were significantly lower in IRS2KO mice. However, similar analysis performed at 28 days post-BDL – when fibrosis is established in this experimental model (Tag et al., 2015) – revealed that serum bilirubin content, as well as the histological features of liver samples, were comparable in both genotypes. It is important to note that levels of serum ALP and hepatic PAI1 remained lower in IRS2KO mice at this final time period, suggesting that, although there were no differences in the degree of fibrosis, IRS2 deficiency protects against long-term damage induced by bile accumulation. Taken together, these data have evidenced a delay in the progression towards fibrosis in IRS2KO mice after BDL, in agreement with the work of Cadoret et al. (2009) in the IGF1RKO mice. Based on that, we could hypothesize that IRS2 (this study) or IGF1R (Cadoret et al., 2009) deficiency specifically protect against IGF1-mediated HSC activation, as demonstrated here by our *in vitro* experiments silencing *IRS2* in LX2 cells as discussed below. However, we cannot rule out the important contribution of other cytokines/growth factors such as TGFβ or platelet-derived growth factor (PDGF) that are potent triggers of HSC activation by initiating signalling cascades not mediated by IRS2 as a docking protein.

On the other hand, although total levels of bile acids accumulated in the gallbladder at 28 days post-BDL were similar between genotypes, a more detailed analysis revealed substantial differences in the profile of their composition. In fact, in the absence of IRS2, Modica et al. (2010) found a significant decrease in the abundance of CDC, one of the most cytotoxic bile acids owing to its high hydrophobicity. Interestingly, a previous study revealed that CDC dose-dependently induced NLRP3 inflammasome activation and the secretion of the pro-inflammatory cytokine IL1β in macrophages (Gong et al., 2016) and this might explain the lower levels of this cytokine detected in the livers of IRS2KO mice at 7 and 28 days post-BDL. Moreover, the accumulated gallbladder content of TLC, a bile acid with high capacity for generating oxidative stress in the liver (Fuentes-Broto et al., 2010), was also lower in IRS2KO mice compared with the WT mice, along with a decreased expression of *Hmox1*. Of note, the reduced pro-inflammatory profile of the bile acids accumulated in the gallbladder of IRS2KO mice after BDL agrees with their reduced inflammatory features associated with the fibrotic process. Likewise, IRS2KO mice showed a lower induction of two relevant HSC activation markers, αSMA and COL1A1, at 3 and 7 days post-BDL compared with WT mice, suggesting that the delay in the development of the fibrotic process is likely due to a reduction of HSC activation.

In agreement with the data obtained in the mouse model, the experiments performed in human LX2 cells with *IRS2* gene silencing showed both prevention of the acquisition of a fibroblast-like phenotype and a significant decrease in the expression of HSC activation markers after the treatment with IGF1. In fact, IGF1-stimulated LX2 cells showed their characteristic activated phenotype and expressed the activation markers COL1A1 and αSMA in comparison with untreated cells. There are controversial results on the capacity of IGF1 to activate HSCs. On the one hand, it has been shown that IGF1 acts on HSCs and suppresses their activation, inducing cellular senescence and preventing fibrosis (Nishizawa et al., 2016). On the other hand, other studies have demonstrated that, in both primary cultures and HSC cell lines, IGF1 increases proliferation (Scharf et al., 1998) and favours the expression of collagen (Svegliati-Baroni et al., 1999). Interestingly, the latter study revealed that insulin induces the proliferation of HSCs, but not collagen synthesis. Therefore, IGF1 is able to induce ERK1/2 phosphorylation in HSCs, an effect that insulin does not achieve in these cells and could be responsible for the IGF1 specificity in increasing collagen levels. In fact, it was reported that the selective MEK inhibitor PD98059 impaired HSC activation (Svegliati-Baroni et al., 1999) in agreement with the results shown in the present study. Notably, inhibition of AKT with LY294002 did not affect the activation of HSCs, suggesting a cellular specificity of the IRS2–ERK1/2 pathway in IGF1-mediated HSC activation.

It is known that ERK1/2 activation increases the expression of MMP9, a metalloprotease involved in degradation of the extracellular matrix (Duarte et al., 2015; Hamada et al., 2008) whose promoter contains AP1 binding domains (Loesch et al., 2010). Several studies have reported elevations of this metalloprotease during the fibrotic process in livers from both humans and rodents (Arthur, 2000), suggesting a pivotal role of ERK1/2 in its induction in HSCs (Gong et al., 2017; Loesch et al., 2010). Analysis of MMP9 expression revealed a significant increase in LX2 cells treated with IGF1; an effect that was abolished by the presence of PD98059 or in *IRS2*-silenced cells in which ERK1/2 phosphorylation was decreased. Thus, these experimental approaches point to the involvement of IRS2 and ERK1/2 in the induction of MMP9 expression and, ultimately, in the activation of HSCs in response to IGF1.

**Fig. 8.**
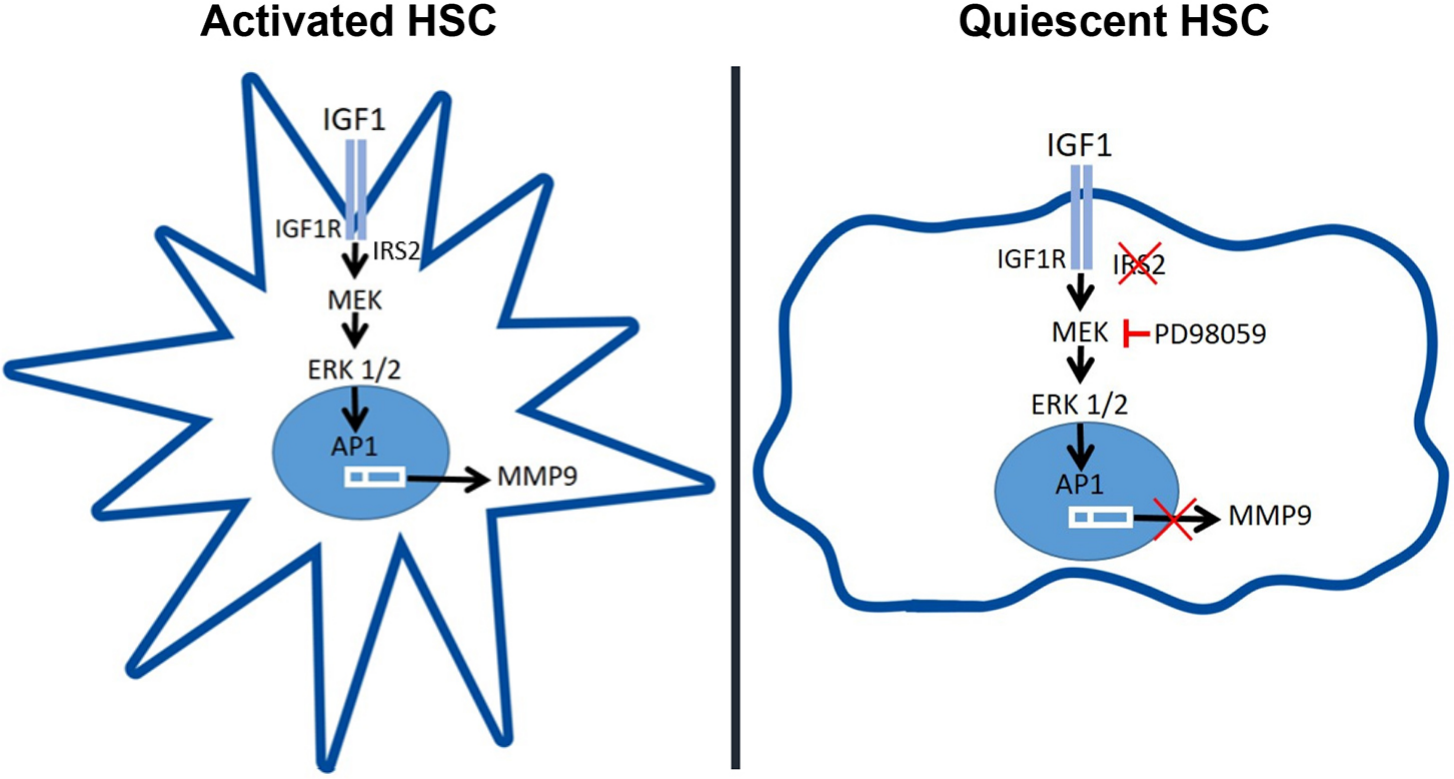
**Lack of IRS2 reduces hepatic MMP9 expression under cholestatic damage.** Schematic diagram of the proposed mechanism by which IGF1 binds to its receptor (IGF1R) in HSCs and signals through IGF1R via IRS2 to trigger ERK1/2 phosphorylation, leading to the expression of MMP9.

According to our proposal of IGF1R–IRS2–ERK1/2–MMP9 as a key axis in regulating HSC activation (Fig. 8), a previous study mentioned above has reported that the lack of IGF1R in liver cells decreases hepatocellular damage and fibrosis in mice at 3 days post-BDL (Cadoret et al., 2009). Interestingly, similarly to our study, the differences between genotypes disappeared at later times post-BDL surgery when the fibrosis was already established. Altogether, the results in two different, but closely related, animal models (IGF1RKO and IRS2KO) strongly suggest that a decrease in the signalling regulated by the IGF1-IGF1R system in HSCs, due to either IGF1R or IRS2 deficiency, delays the fibrotic process induced by cholestatic damage. Moreover, the lower induction of *Mmp9* gene expression observed in the livers from IRS2KO mice after BDL suggests that IGF1R–IRS2–ERK1/2–MMP9 signalling might be attenuated in these mice compared with their corresponding WT counterparts, thereby elucidating a potential molecular mechanism responsible for this effect.

In conclusion, this study provides new mechanistic evidence to suggest that therapies aimed at increasing IGF1 action in liver cells to counteract acute and/or chronic liver damage need to be carefully evaluated in order to avoid secondary undesired effects due to its ability, possibly acting in concert with other cytokines and growth factors, of maintaining persistent HSC activation.

## MATERIALS AND METHODS

### Animals

Mice were housed in light/dark (12 h light/12 h dark), temperature (22°C)- and humidity-controlled rooms, fed standard chow diet *ad libitum* and had free access to drinking water at the animal facilities of the Instituto de Investigaciones Biomédicas Alberto Sols (CSIC-UAM, Madrid). All animal experimentation was conducted according to Spanish and European legislation and approved by the CSIC and Comunidad de Madrid Animal Care and Use Committees. Wild-type (WT) and *Irs2*-deficient (IRS2KO) mice were maintained on a similar mixed genetic background (C57BL/6×129sv) as previously described (González-Rodriguez et al., 2010, 2015; Withers et al., 1998).

### Bile duct ligation

Female animals at 10- to 12-weeks of age were submitted to BDL surgery, which consisted of a double ligation with a 4-0 silk suture and section between the two ligatures of the common bile duct under isoflurane anaesthesia (Tag et al., 2015). As control animals, mice were submitted to sham operations that consisted in laparotomy followed by a careful bile duct exposure without ligation (*n*=8 animals per genotype). Mice were sacrificed by decapitation after 3 (*n*=8 animals per genotype), 7 (*n*=6 animals per genotype) or 28 (*n*=8 animals per genotype) days post-BDL. Serum samples and livers were collected and frozen at −80°C or fixed with 4% paraformaldehyde for further analysis.

### Histopathology assessment

Paraffin-embedded liver sections (4 µm) were stained with haematoxylin and eosin (H&E), Masson's trichrome solution or Sirius Red, and evaluated by an experienced liver pathologist who assessed the fibrosis stage using the system for mouse models validated by Liang et al. (2014), which defined fibrosis staging as: 0, none; 1, perisinusoidal and/or pericentral; 2, incomplete central/central bridging fibrosis; 3, complete central/central bridging fibrosis.

### Serum analysis

Bilirubin and alkaline phosphatase (ALP) serum levels were determined using Reflotron strips (Roche Diagnostics, Barcelona, Spain) following the manufacturer's instructions.

### Bile acid analysis in the gallbladder

Bile fluid was directly extracted from the gallbladder with a syringe soon after death and immediately transferred to an Eppendorf tube, which was stored at −80°C in order to preserve the bile acids. Samples were analyzed at CEMBIO metabolomic unit (Universidad San Pablo CEU, Madrid, Spain). The analysis was carried out according to the methodology described previously (Alnouti et al., 2008) with slight modifications. After diluting samples (1:100 in distilled water), bile acids were extracted by solid phase extraction (SPE) using the Oasis HLB 3 cc 60 mg 30 μm cartridges (Waters, Milford, MA, USA) as adsorbent. The cartridges were conditioned with methanol:water (1:1) and after loading the sample, they were washed with water and the bile acids were eluted with methanol. The solvent was then evaporated and the samples were resuspended in methanol:water (1:1). The content of bile acids in the bile fluid was determined by UHPLC (ultra-high-performance liquid chromatography) using a 1290 Infinity II LC system connected to a MS QqQ 6460 as detector, both from Agilent Technologies (Santa Clara, CA, USA). For separation, a Zorbax Eclipse Plus C8 2.1×150 mm 1.8 μm column (Agilent Technologies) and an optimized gradient with acetonitrile, methanol and ammonium acetate (pH 4) were used. Commercial standards (Sigma-Aldrich, Madrid, Spain) were used for all measured bile acids and D4-deoxycholic acid as internal standard (PI). The calibration curves were prepared with seven concentrations of each standard.

### Isolation of HSCs from mice

HSCs were isolated by collagenase–pronase perfusion of sham-operated livers or 3 days after BDL as described originally by Friedman and Roll, with the minor modifications introduced by Rippe and co-workers (Friedman and Roll, 1987; Rippe et al., 1995). Once isolated, HSCs were immediately resuspended in TRIzol reagent (Vitro, Sevilla, Spain) for RNA extraction.

### Cell culture and treatments

The human HSC line LX2 was purchased from the American Type Culture Collection (ATCC, Manassas, VA). Cells were maintained in Dulbecco's modified Eagle's medium (DMEM, Invitrogen, Barcelona, Spain) containing high glucose, penicillin/streptomycin and 2% fetal bovine serum. LX2 cells were treated with 10 nM IGF1 (Preprotech, London, UK) for different periods. For the inhibition of ERK1/2 or AKT, LX2 cells were pre-treated with 20 µM PD98059 or 25 µM LY294002 (Sigma-Aldrich), respectively, for 2 h prior to stimulation with IGF1.

### Immunofluorescence and confocal imaging

LX2 cells were grown on glass coverslips and treated as described in the Results and figure legends. Then, cells were washed twice with phosphate buffered saline (PBS), fixed in 4% paraformaldehyde for 10 min and processed for immunofluorescence with the indicated primary antibodies: anti-αSMA (A-2547) purchased from Sigma-Aldrich, anti-COL1A1 (sc-8784) and anti-MMP9 (sc-6840) from Santa Cruz Biotechnology (Santa Cruz, CA, USA) and the appropriate FITC-conjugated secondary antibodies [Alexa Fluor^®^ 488 goat anti-rabbit IgG (A11034) or Alexa Fluor^®^ 488 goat anti-mouse IgG (A11001), Life Technologies, Grand Island, NY, USA]. Mounting medium used was Fluoromont G^®^ (BioNova cientifica, Madrid, Spain). Immunofluorescence was examined using a confocal microscope (Leica TCS SP5 X, Barcelona, Spain) and image analysis procedures were performed with Fiji software (NIH, Bethesda, MD).

### Gene expression analysis by real-time quantitative PCR

Total RNA from cells or liver samples was extracted with TRIzol reagent and was reverse transcribed using a reverse transcription system (Promega Inc., Madrid, Spain) in a T100TM Thermal Cycler (BioRad Inc., Madrid, Spain) following the manufacturer's instructions. Real-time quantitative polymerase chain reaction (RT-qPCR) was performed with StepOnePlus^TM^ Real Time PCR System sequence detector (Thermo Fisher Scientific, Inc., Madrid, Spain) with the SYBR Green method and d(N)6 random hexamer with primers purchased from (Metabion, Steinkirchen, Germany). Each sample was run in duplicate and normalized to GAPDH gene expression. Fold changes were determined using the ΔΔCt method. Primer sequences are listed in Table S1.

### Preparation of protein extracts and western blotting

To obtain total cell lysates, at the end of the experiment attached cells were scraped off and incubated for 10 min on ice with lysis buffer (50 mM Tris-HCl, pH 7.4, 1% Triton X-100, 0.2% SDS, 1 mM EDTA, 1 mM PMSF and 5 µg/ml leupeptin). After protein content determination with Bradford reagent, total protein was boiled in Laemmli sample buffer and separated by 8% SDS-PAGE. Proteins were then transferred to Immunoblot nitrocellulose membranes (Bio-Rad) and, after blocking, incubated overnight with the indicated primary antibodies: anti-AKT (9272), anti-phospho ERK1/2 (9101), anti-ERK1/2 (9102) and anti-phospho IGF1R (3021) from Cell Signaling Technology (Boston, MA, USA), anti-phospho IRS1 (07-844) from Merk Millipore (Darmstadt, Germany) and anti-phospho AKT (sc-7985-R) and anti-IGF1R (sc-713) from Santa Cruz Biotechnology. Immunoreactive bands were visualized using the ECL Western blotting protocol (Bio-Rad).

### Short hairpin IRS2 knockdown

Human scrambled (shControl, shC) and IRS2 shRNA lentiviral particles (Sigma-Aldrich) were used to produce stable IRS2 knockdown in LX2 cells. Proliferating cells were co-incubated with lentiviral transducing particles in culture medium containing 8 μg/μl hexabromide (Santa Cruz) for 24 h, and then cultured with 5–10 µg/ml puromycin (Santa Cruz). Resistant cells were expanded and examined for *IRS2* mRNA levels.

### Statistical analysis

Mice were randomly assigned to each experimentation group. The number of animals necessary to detect a standardized effect size was determined via *a priori* sample size calculation with the following assumptions: α=0.05, β=0.2, mean=20% standard deviation of the mean. Data analysis was performed in a blinded manner. Once data were tested with a Shapiro–Wilk test for normality and Levene's test for equality of variance, data from two groups were compared by using unpaired two-tailed Student's *t*-test. When more than two groups were involved, data were compared by using one-way analysis of variance (ANOVA) followed by Bonferroni test. All statistical analyses were performed using GraphPad (San Diego, CA, USA) Prism 7.0 software with two-sided tests and a *P*-value of <0.05 was considered statistically significant. Data from RT-qPCR experiments are expressed as fold increase and presented as mean±s.e.m. relative to control condition. Data from immunofluorescence analysis are expressed as fold increase and presented as mean±s.e.m. relative to control condition. Data from western blot experiments are expressed as percentage of IGF1 stimulation and presented as mean±s.e.m. relative to control condition (100%).

## Supplementary Material

Supplementary information
