# Supplementary material for: Insulin receptor substrate 2 (IRS2) deficiency delays liver fibrosis associated with cholestatic injury
Source: Dis Model Mech. 2019 Jul 16;12(7):dmm038810. doi: 10.1242/dmm.038810 (PMC6679376; doi:10.1242/dmm.038810)
Supplement: Supplementary information [file dmm-12-038810-s1.pdf]

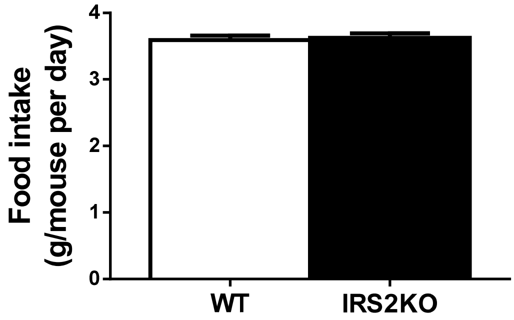

Figure S1. Mean daily food intake. Experimental groups: Wild-type (WT) and *Irs2*-deficient (IRS2KO) female mice (n=8 animals per condition).

Disease Models & Mechanisms • Supplementary information

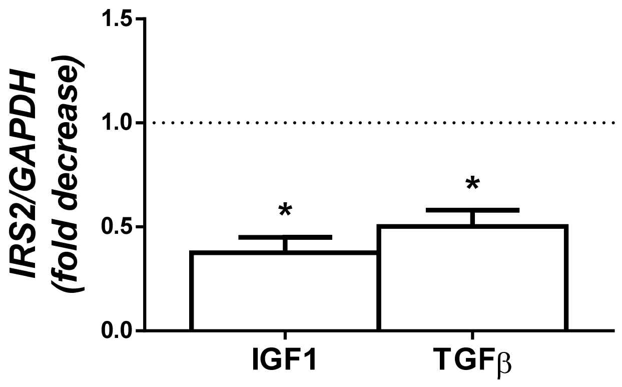

Figure S2. LX2 cells were treated with 10 nM IGF1 or 5 ng/ml TGFβ for 24 h. *IRS2* mRNA levels determined by RT-qPCR. Statistical significance was carried out by unpaired two-tailed Student's t-test: *p<0.05, IGF1 or TGFβ vs. untreated cells (n=3 independent experiments performed in duplicate).

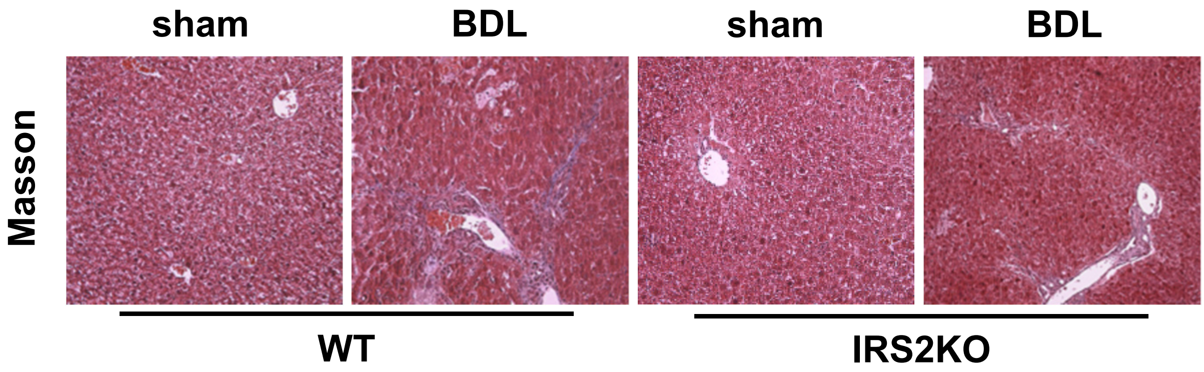

Figure S3. Representative images (20x) from Masson`s trichrome staining. Experimental groups: Wild-type (WT) and *Irs2*-deficient (IRS2KO) female mice submitted to BDL and analyzed 28 days after surgery (n=8 animals per condition).

Disease Models & Mechanisms • Supplementary information

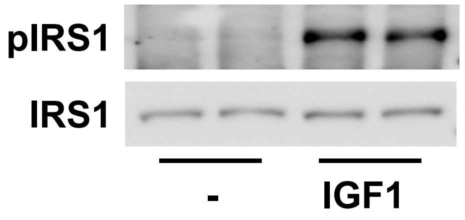

Figure S4. LX2 cells were treated with 10 nM IGF1 for 15 min and IRS1 tyrosine phosphorylation was analyzed by Western Blot. Representative images are shown (n=3 independent experiments performed in duplicate).

Disease Models & Mechanisms • Supplementary information

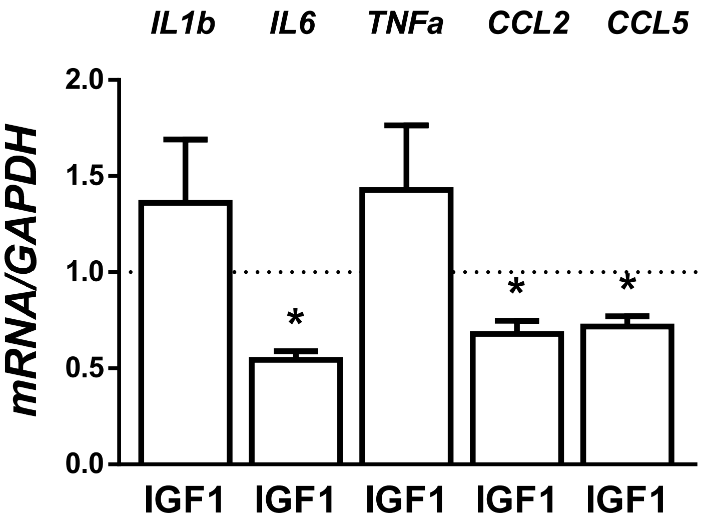

Figure S5. LX2 cells were treated with 10 nM IGF1 for 24 h. mRNA levels of pro-inflammatory cytokines determined by RT-qPCR. Statistical significance was carried out by unpaired two-tailed Student's t-test: *p<0.05, IGF1 vs. untreated cells (n=3 independent experiments performed in duplicate).

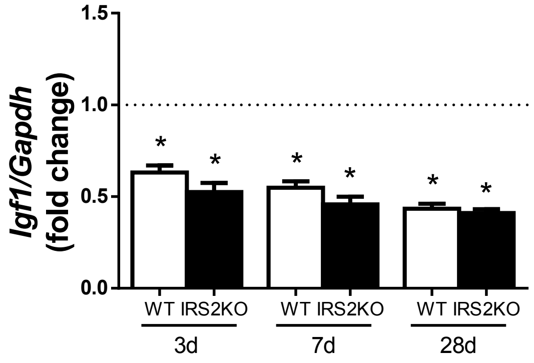

Figure S6. *Igf1* mRNA levels determined by RT-qPCR. Experimental groups: Wild-type (WT) and *Irs2*-deficient (IRS2KO) female mice submitted to BDL and analyzed at the indicated time-periods. Statistical significance was carried out by one-way ANOVA with Bonferroni post-test: *p<0.05, BDL vs. Sham (n=6-8 animals per condition).

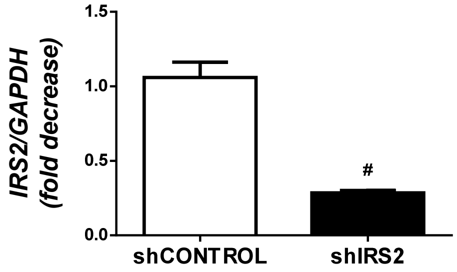

Figure S7. LX2 cells infected with scrambled (shC) or IRS2 shRNA (shIRS2) lentiviral particles. *IRS2* mRNA levels determined by RT-qPCR. Statistical significance was carried out by unpaired two-tailed Student's t-test: #$p<0.05$, shIRS2KO vs. shC (n=4 independent experiments performed in duplicate).

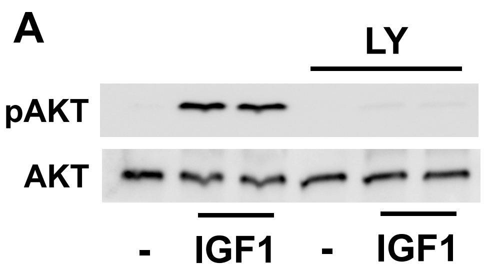

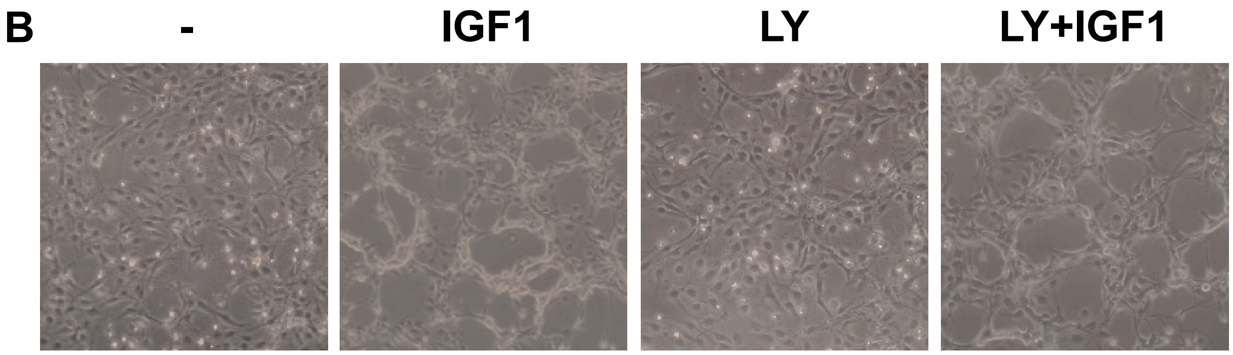

Figure S8. LX2 cells were pretreated with 25 μM LY294002 for 2 h prior to 10 nM IGF1 stimulation for 15 min (A) or 24 h (B). **A**. Representative Western blot with the indicated antibodies. **B**. Representative phase-contrast images. (n=3 independent experiments performed in duplicate).

Table S1. *Primer sequences for RT-qPCR.*

m-Gapdh	Forward<br>Reverse	TGGAAGAGTGGGAGTTGCTGT<br>CCTGGAGAAACCTGCCAAGTA
m-Acta2	Forward<br>Reverse	CCCAGACATCAGGGAGTAATGG<br>TCTATCGGATACTTCAGCGTCA
m-Mmp9	Forward<br>Reverse	AAACCTTCAAAGGCCTCAAGTG<br>GTCTCGCGGCAAGTCTTCAG
m-Pai1	Forward<br>Reverse	ATGACTGGGTGGAAAGGCATAC<br>CAGGCGTGTCAGCTCGTCTA
m-Hmox1	Forward<br>Reverse	CACAGATGGCGTCACTTCGTC<br>GTGAGGACCCACTGGAGGAG
m-Il6	Forward<br>Reverse	GAGGATACCACTCCCAACAGACC<br>AAGTGCATATCGTTGTTCATACA
m-Il1b	Forward<br>Reverse	AGAAGCTGTCGCAGGTACCTG<br>GGAAAAGAAGGTGCTCATGTCC
m-Tnfa	Forward<br>Reverse	CATCTTCTCAAAATTCGAGTGACAA<br>TGGGAGTAGACAAGGTACAACCC
m-Irs2	Forward<br>Reverse	CACGAGCCCGTAGTTGTCAT<br>ACCGACTTGGTCAGCGAAG
m-Col1a1	Forward<br>Reverse	TAGGCCATTGTGTATGCAGC<br>ACATGTTCAGCTTTGTGGACC
m-Igf1	Forward<br>Reverse	TCAACAAGCCCACAGGCTATG<br>GCTCCGGAAGCAACACTCAT
h-IRS2	Forward<br>Reverse	CAGTGCTGAGCGTCTTCTTTT<br>ACCTACGCCAGCATTGACTT
h-IL1b	Forward<br>Reverse	TGAGCACCTTCTTTCCCTTCA<br>GTAGTGGTGGTCGGAGATTCG
h-IL6	Forward<br>Reverse	CCTGACCCAACCACAAATGC<br>CCTTAAAGCTGCGCAGAATGA
h-TNFA	Forward<br>Reverse	TCGAACCCCGAGTGACAAG<br>TTGGCCAGGAGGGCATT
h-CCL2	Forward<br>Reverse	GAAGAATCACCAGCAGCAAGTG<br>CAGATCTCCTTGGCCACAATG
h-CCL5	Forward<br>Reverse	CGTGCCCACATCAAGGAGTA<br>CGGTTCTTTCGGGTGACAAA
h-GAPDH	Forward<br>Reverse	GAAGGTGAAGGTCGGAGT<br>CATGGGTGGAATCATATTGGAA

Disease Models & Mechanisms • Supplementary information
